# S3-3 Effects of a physical literacy intervention delivered in a medical center setting

**DOI:** 10.1093/eurpub/ckad133.016

**Published:** 2023-09-11

**Authors:** Jean-Pierre Weerts, Alexandre Mouton

**Affiliations:** Sport Sciences Department, University of Liege, Belgium; Sport Sciences Department, University of Liege, Belgium

## Abstract

**Purpose:**

The concept of Physical Literacy (PL) suggests that an individual with a holistic vision of physical activity will have more chances to remain physically active over life (Longmuir & Tremblay, 2016). Following the development of an assessment tool for PL specifically targeting an adult population with chronic disease, our study sought to find out the effect of successive PL interventions by a physical educator in a medical center on the PL level of patients.

**Methods:**

A quantitative, within-subject design was conducted. PL program adhesion was promoted by doctors or physiotherapists of the medical center. Volunteered patients took part in two PL assessments and counseling meetings with a specialized physical educator. Following a general anamnesis and the Ricci-Gagnon physical activity questionnaire, PL scores were calculated based on a new assessment tool design, consisting of a 40-item questionnaire and 4 physical tests; and divided into the 4 domains of PL (psychological, social, cognitive, physical). With the intervention being electronically completed during the assessment, feedback was given immediately to the patient. Following a motivational interviewing technique, results were discussed with the patient leading towards appropriate SMART individual goals to be set.

**Results:**

A total of 108 patients with at least one chronic disease (71% female, 56±15 years) underwent assessment 1, with 46 patients (74% female, 61±13 years) completing assessment 2 (46 days later on average). Significant improvements were demonstrated for the overall PL score (p < 0,001) as well as the cognitive (p = 0,001) and physical domains (p = 0,032). No significant changes were found for the psychological and social domain. Additionally, no significant difference in changes were found based on age, sex or nature of the chronic disease.

**Conclusions:**

This pilot-study was a first attempt to measure and evaluate the significance of changes in PL scores for a population of adults with chronic disease. If validation of this developed assessment tool requires more research, results show promising effect of an intervention by ongoing support of PL on the domains and overall scores of PL.

**Funding Source:**

This study received no external funding.

